# The SIRT1/Nrf2 signaling pathway mediates the anti-pulmonary fibrosis effect of liquiritigenin

**DOI:** 10.1186/s13020-024-00886-1

**Published:** 2024-01-18

**Authors:** Qingzhong Hua, Lu Ren

**Affiliations:** 1 The Second Affiliated Hospital of Shenzhen University (People’s Hospital of Shenzhen Baoan District), Shenzhen, 518101 Guangdong China; 2 Clinical Nursing Teaching and Research Section, The Second Xiangya Hospital, Central South University, Changsha, 410011 Hunan China; 3 Department of Dermatology, The Second Xiangya Hospital, Central South University, Changsha, 410011 Hunan China

**Keywords:** Liquiritigenin, SIRT1, Nrf2, Oxidative stress, Myofibroblast differentiation, Pulmonary fibrosis

## Abstract

**Background:**

At present, the treatment options available for idiopathic pulmonary fibrosis are both limited and often come with severe side effects, emphasizing the pressing requirement for innovative therapeutic alternatives. Myofibroblasts, which hold a central role in pulmonary fibrosis, have a close association with the Smad signaling pathway induced by transforming growth factor-β1 (TGF-β1) and the transformation of myofibroblasts driven by oxidative stress. Liquiritigenin, an active compound extracted from the traditional Chinese herb *licorice*, boasts a wide array of biomedical properties, such as anti-fibrosis and anti-oxidation. The primary objective of this study was to examine the impact of liquiritigenin on bleomycin-induced pulmonary fibrosis in mice and the underlying mechanisms.

**Methods:**

The anti-pulmonary fibrosis and anti-oxidant effects of liquiritigenin in vivo were tested by HE staining, Masson staining, DHE staining and bio-chemical methods. In vitro, primary mouse lung fibroblasts were treated with TGF-β1 with or without liquiritigenin, the effects of liquiritigenin in inhibiting differentiation of myofibroblasts and facilitating the translocation of Nrf2 were valued using Quantitative real-time polymerase chain reaction (Q-PCR), western blotting and immunofluorescence. Nrf2 siRNA and SIRT1 siRNA were used to investigate the mechanism underlies liquiritigenin’s effect in inhibiting myofibroblast differentiation.

**Results:**

Liquiritigenin displayed a dose-dependent reduction effect in bleomycin-induced fibrosis. In laboratory experiments, it was evident that liquiritigenin possessed the ability to enhance and activate sirtuin1 (SIRT1), thereby facilitating the nuclear translocation of Nrf2 and mitigating the oxidative stress-induced differentiation of primary mouse myofibroblasts. Moreover, our investigation unveiled that SIRT1 not only regulated myofibroblast differentiation via Nrf2-mediated antioxidant responses against oxidative stress but also revealed liquiritigenin's activation of SIRT1, enabling direct binding to Smad. This led to decreased phosphorylation of the Smad complex, constrained nuclear translocation, and suppressed acetylation of the Smad complex, ultimately curtailing the transcription of fibrotic factors. Validation in live subjects provided substantial evidence for the anti-fibrotic efficacy of liquiritigenin through the SIRT1/Nrf2 signaling pathway.

**Conclusions:**

Our findings imply that targeting myofibroblast differentiation via the SIRT1/Nrf2 signaling pathway may constitute a pivotal strategy for liquiritigenin-based therapy against pulmonary fibrosis.

## Introduction

Idiopathic Pulmonary Fibrosis (IPF) is a chronic, progressive lung disease characterized by the accumulation of myofibroblasts and extensive deposition of extracellular matrix (ECM) within the lung tissue: myofibroblasts in the fibrotic foci secret excessive extracellular matrix, mainly collagens, leading to scarring and destruction of lung architecture [1]. The median survival time post-diagnosis is only 3 years [2]. Currently, pharmacological treatment options for IPF are suboptimal. While pirfenidone and nintedanib have been approved for IPF therapy, their effectiveness is limited, and concerns regarding their safety persist [3]. Therefore, the development of safe and efficacious therapeutic drugs is of paramount importance for ameliorating patient symptoms and reducing IPF mortality.

Reactive oxygen species (ROS), the typical byproducts of oxygen metabolism, are vital for cell metabolism and intracellular signaling [4]. Specific pathological triggers can significantly elevate ROS levels, leading to extensive cell damage and functional impairments, resulting in oxidative stress [5, 6]. Oxidative stress is considered a crucial molecular mechanism associated with fibrotic diseases, increasing evidence indicates that oxidative stress contributes to the processes of pulmonary fibrosis, including myofibroblast differentiation, alveolar epithelial cell apoptosis, endothelial cell barrier disruption, and elevated expression of transforming growth factor-beta 1 (TGF-β1), promoting fibrosis [7–9]. Further, deletion of antioxidant defenses leads to oxidative stress, augments fibrosis [7]. To prevent damage from oxidative stress, many substances in the body participate in antioxidant defense, with nuclear factor erythroid 2-related factor 2 (Nrf2) being a key regulatory factor [10]. In a bleomycin-induced pulmonary fibrosis mouse model, Nrf2 knockout mice exhibited increased sensitivity to bleomycin-induced lung fibrosis compared to wild-type mice [11]. Additionally, primary lung fibroblast cultures obtained from control and IPF patients suggested that reduced Nrf2 expression was associated with increased myofibroblast differentiation, while Nrf2 activation enhanced antioxidant defense and reduced myofibroblast phenotypic expression [12]. These findings suggest that Nrf2 may serve as a therapeutic target for IPF and other fibrotic diseases induced by oxidative stress.

As an upstream kinase of Nrf2, Sirtuin1 (SIRT1) is a cytosolic nicotinamide adenine dinucleotide (NAD +)-dependent deacetylase [13–15]. Upon activation, it promotes the nuclear translocation of Nrf2 and enhances Nrf2's nuclear transcription by deacetylating Nrf2 [15, 16]. SIRT1 levels are suppressed in pulmonary fibrosis lesions [17–19]. Additionally, SIRT1 overexpression has been demonstrated to improve age-related pulmonary fibrosis [20]. Resveratrol, a SIRT1 activator, has the potential to alleviate cardiac, hepatic, and renal fibrosis [21–23]. While the significant role of SIRT1 in fibrosis has been confirmed, its precise mechanism of action remains unclear. Only a few small molecules targeting SIRT1 have been studied for the treatment of pulmonary fibrosis. Therefore, there is an urgent need to develop drug strategies that utilize SIRT1 for fibrosis treatment.

Liquiritigenin, an active ingredient derived from the dual-purpose medicinal herb *licorice*, is a compound obtained from its herbal extract. It has been shown to possess significant immunomodulatory, anti-allergic, and anti-inflammatory biological effects [24–27]. However, the impact and mechanisms of action of liquiritigenin on pulmonary fibrosis remain unclear. In this study, we have made the novel discovery that liquiritigenin can activate and promote the expression of SIRT1, which, in turn, exerts antioxidant capabilities by modulating Nrf2, thereby inhibiting myofibroblast differentiation involved in oxidative stress. Additionally, we found that SIRT1 activated by liquiritigenin can directly interact with the Smad complex, inhibiting the phosphorylation of Smad3 and Smad4 induced by TGF-β1, thus preventing their nuclear translocation and also blocking the acetylation of Smad3 and Smad4, further inhibiting their nuclear transcription. This study aims to investigate the effects and mechanisms of liquiritigenin on bleomycin-induced pulmonary fibrosis in vivo and TGF-β-induced myofibroblast differentiation in vitro.

## Materials and methods

### Experimental animals

Female C57BL/6 mice (6–8 weeks old) were housed under specific pathogen-free (SPF) conditions with unrestricted access to food and water. This study adhered to ethical principles for experimental animal welfare.

To evaluate the therapeutic effects of liquiritigenin, mice were intratracheally injected with 50 μL of bleomycin (3 mg/kg, diluted in 0.9% saline) (Nippon Kayaku, Japan) or with an equivalent volume of 0.9% saline on day 0 following pentobarbital sodium anesthesia. Liquiritigenin (Selleck, China) was diluted in 0.9% saline and subjected to ultrasonication. A total of 75 mice were randomly divided into five groups and treated as follows: control group (intratracheal 0.9% saline on day 0 + oral 0.9% saline on days 15–28); pulmonary fibrosis model group (intratracheal bleomycin on day 0 + oral 0.9% saline on days 15–28); low does group (intratracheal bleomycin on day 0 + oral liquiritigenin 25 mg/kg on days 15–28); medium does group (intratracheal bleomycin on day 0 + oral liquiritigenin 50 mg/kg on days 15–28); high does group (intratracheal bleomycin on day 0 + oral liquiritigenin 100 mg/kg on days 15–28).

The dose (100 mg/kg) that resulted in the most effective in the treatment of fibrosis was chosen for further investigation of the molecular mechanism of liquiritigenin. Seventy-five mice were randomly divided into five groups: control group (intratracheal 0.9% saline on day 0 + oral 0.9% saline on days 15–28); pulmonary fibrosis model group (intratracheal bleomycin on day 0 + oral 0.9% saline on days 15–28); liquiritigenin treatment group (intratracheal bleomycin on day 0 + oral liquiritigenin 100 mg/kg on days 15–28); SIRT1 inhibitor group (intratracheal bleomycin on day 0 + intraperitoneal EX527 10 mg/kg on days 15–28 + oral liquiritigenin 100 mg/kg on days 15–28); Nrf2 inhibitor group (intratracheal bleomycin on day 0 + intraperitoneal ML385 25 mg/kg on days 15–28 + oral liquiritigenin 100 mg/kg on days 15–28). ML385 and EX527 were administered intraperitoneally, 3 h before oral administration of liquiritigenin. On day 29, the mice were euthanized for lung tissue collection.

### Histological analysis

The lung tissues were fixed in a 4% (w/v) paraformaldehyde solution, followed by paraffin embedding and sectioning. These sections were subsequently subjected to hematoxylin–eosin and Masson's trichrome staining.

### Hydroxyproline assay

We utilized a Hydroxyproline Assay Kit (Nanjing Institute of Built-up Biotechnology, Nanjing, China) to determine the hydroxyproline content in lung tissue. Lung tissue fragments were homogenized in a solution at a ratio of 1 mg tissue to 9 ml solution (w/v). The homogenized samples were then mixed with the digestion solution from the kit at a volume ratio of 5:1 (v/v), resulting in a total volume of 300 μL. This mixture was incubated in a water bath at 37 °C for 3 h. Following this incubation, 500 μL of reagent 1 was added to the sample and allowed to stand at room temperature for 10 min. Subsequently, 500 μL of reagent 2 was introduced, and the mixture was left at room temperature for 5 min. Finally, 1 mL of reagent 3 was added, thoroughly mixed, and incubated in a water bath at 60 °C for 15 min. After cooling, the mixture underwent centrifugation at 3500 rpm for 10 min, and the supernatant was meticulously collected without disturbing the sediment at the bottom. The absorbance at 550 nm was measured in each tube using a cuvette with a 1 cm path length, and zero adjustment was performed using double-distilled water.

### Immunofluorescence

Immunofluorescence was employed to examine the expression and localization of α-SMA, Collagen I, and SIRT1 in primary lung myofibroblasts. After the samples were fixed in a 4% paraformaldehyde solution, they underwent washing and permeabilization using a 0.5% Triton X-100 solution. Subsequently, to block non-specific binding, goat serum blocking solution (ZSGB Biologicals, China) was applied. Primary antibodies, including Collagen I polyclonal antibody (1:100, Proteintech, China), α-SMA polyclonal antibody (1:100, Proteintech, China), and SIRT1 polyclonal antibody (1:100, Proteintech, China), were incubated with the samples overnight at 4 °C. Following a thorough wash with PBST, fluorescent secondary antibodies (1:100; Proteintech, China) diluted in PBS containing 0.1% (v/v) Tween 20 were applied. This mixture was then incubated at room temperature for 1 h, and DAPI staining was performed to label the cell nuclei. Finally, the sections were treated with an antifading agent (Solarbio, China) and covered with a mounting medium.

### Cells culture

Primary mouse lung fibroblasts were obtained through a digestion method. In brief, mice were anesthetized using sodium pentobarbital, and their lungs were excised, perfused with pre-chilled PBS, and sectioned into 1–2 cm^2^ pieces. The lung tissue fragments were then digested in Dulbecco's modified Eagle medium (DMEM) digestion medium containing 1 mg/ml collagenase I at 37 °C for 1 h. After digestion, the cells were passed through 70 μm and 40 μm cell filters, centrifuged, and resuspended in high-glucose DMEM (Gibco, Waltham, MA, USA) supplemented with 20% fetal bovine serum (FBS, Sigma, USA) and 1% streptomycin/penicillin (Gibco, Waltham, MA, USA). These cells were cultured at 37 °C in a humidified atmosphere with 5% CO2.

Upon reaching a confluence of over 90%, the cells were trypsinized using 0.5% w/v trypsin–EDTA (Hyclone) for passaging. After three purification rounds, the cells were ready for subsequent experiments. Subsequently, primary mouse fibroblasts were induced into myofibroblasts by stimulating them with TGF-β1 (10 ng/mL, Peprotech, USA) for 48 h. Liquiritigenin intervention was applied 2 h prior to TGF-β1 treatment to investigate the impact and mechanism of liquiritigenin on TGF-β1-induced myofibroblast transformation.

### Cell viability assay

Cell viability was evaluated utilizing the Cell Counting Kit-8 (CCK-8) (Biosharp, China). Cells were plated onto 96-well plates and exposed to different concentrations of liquiritigenin (0, 1, 3, 10, 30, and 100 μM). Following 48 h of TGF-β1 treatment, a high-glucose DMEM solution containing 10% CCK-8 was introduced into each well, and incubation was carried out at 37 °C for 1.5 h. The absorbance was then measured at 450 nm.

### RNA extraction and Quantitative real-time polymerase chain reaction (Q-PCR)

Total RNA was isolated from lung tissue or cells using TRIzol Reagent (Thermo Fisher Scientific, USA). Subsequently, cDNA was synthesized using a Reverse Transcription Kit from Thermo Fisher Scientific. Quantitative PCR (Q-PCR) was carried out with SYBR GREEN (Promega, USA) on a Bio-Rad CFX96 Touch Real-Time PCR Detection System (Bio-Rad, USA). The Q-PCR protocol consisted of an initial denaturation step at 95 °C for 2 min, followed by 40 cycles of 95 °C for 3 s and 60 °C for 30 s. The procedure concluded with a melting curve analysis spanning from 60 °C to 95 °C. Primer sequences are followed: β-actin, forwrad-5′-CCTGCGACTTCAACAGCAAC-3′, reverse-5′-TGGGATAGGGCCTCTCTTGC-3′; Collagen I, forward-5′-GAGCGGAGAGTACTGGATCG-3′, reverse-5′-GCTTCTTTTCCTTGGGG-TTC-3′; α-SMA, forward-5′-GCGTGGCTATTCCTTCGTGACTAC-3′, reverse-5′-CGTCAGGCAGTTCGTAGCTCTTC-3′; SIRT1, forward-5′-ATGCCAGAGTCCAAGTTTAGAAGAACC-3′, reverse-5′-AAATCCAGATCCTCCAGCACATTCG-3′.

### Western blotting

Lung tissues and cells were lysed using radioimmunoprecipitation assay (RIPA) lysis buffer from Solarbio, China. The protein concentration was determined using the bicinchoninic acid (BCA) assay. A 10% fraction of the total protein was separated by electrophoresis and transferred onto PVDF membranes (Millipore, USA). These membranes were then blocked in a solution consisting of 5% skim milk in a TBS buffer containing 0.1% Tween 20 (TBST).

Next, the membranes were incubated with primary antibodies overnight at 4 °C, including β-actin monoclonal antibody (1:5000, Proteintech, China), α-SMA monoclonal antibody (1:1000, CST, USA), collagen I polyclonal antibody (1:1000, Millipore, USA), SIRT1 polyclonal antibody (1:1000, Proteintech, China), CAT polyclonal antibody (1:3000, Abcam, UK), HO-1 polyclonal antibody (1:1000, Abcam, UK), NQO-1 monoclonal antibody (1:1000, Abcam, UK), Nrf2 polyclonal antibody (1:1000, Proteintech, China), smad3 monoclonal antibody (1:1000, Proteintech, China), Pho-smad3 monoclonal antibody (1:1000, Proteintech, China), smad4 monoclonal antibody (1:1000, Proteintech, China), Pho-smad4 monoclonal antibody (1:1000, Proteintech, China).

Afterward, the membranes were washed three times with TBST and then incubated with either horseradish peroxidase-conjugated goat anti-rabbit immunoglobulin G monoclonal antibody (1:5000, Proteintech, China) or goat anti-mouse immunoglobulin G monoclonal antibody (1:5000, Proteintech, China) for 2 h at room temperature.

Finally, protein bands were visualized using the Luminata™ Crescendo chemiluminescent horseradish peroxidase substrate from Millipore, USA, and the images were captured using a Bio-Rad imager from USA.

### siRNA Transfection

siRNAs specific for SIRT1 (Santa Cruz, USA), Nrf2 (Santa Cruz, USA) or scrambled control (Santa Cruz, USA) were transfected into primary mouse fibroblasts using Lipofectamine™ 3000 (Invitrogen, USA). Knockdown efficiency was determined by Q-PCR and western blotting.

### ROS levels and mitochondrial ROS levels

ROS levels were assessed using H2DCFDA (50 μM, Invitrogen™, USA), while mitochondrial ROS levels were determined using Mitosox (5 μM, Invitrogen, USA), following the manufacturer's instructions. In brief, cells were washed twice with cold PBS and suspended in serum-free DMEM medium at a concentration of 1 × 10^6^ cells/ml. The cells were then stained with H2DCFDA or Mitosox dyes and incubated for 30 min at 37 °C, shielded from light. The results were visualized and captured using fluorescence microscopy (Nikon Ti-s, Tokyo, Japan).

### Immunoprecipitation assay

To assess the interactions between SIRT1 and Smad3, SIRT1 and Smad4, Ac-lysine and Smad3, Ac-lysine and Smad4, we conducted the following experimental steps: Firstly, cells were washed three times with PBS, and then lysed on ice for 1 h in a lysis buffer containing complete protease inhibitor PMSF (Solarbio, China). Subsequently, cells were collected and centrifuged at 12,000 rpm for 10 min at 4 °C. The supernatant was collected and incubated with Dynabeads™ Protein G (Invitrogen, USA) for 3 h at 4 °C, followed by centrifugation. Dynabeads were separated using DynaMag™-2 (Invitrogen, USA). The supernatant was supplemented with antibodies (Proteintech, China) and incubated overnight at 4 °C. Dynabeads were separated again using DynaMag™-2 and washed three times with lysis buffer. The immunoprecipitated proteins were analyzed by western blotting.

### Measurement of MDA, GSH and SOD levels in lung tissues

After euthanizing the mice, the right lungs were removed. Then, we homogenized the lung tissues and prepared them in an extraction buffer for the analysis of MDA, SOD, and GSH contents. To determine the extent of lipid peroxidation in the lung tissue, we measured MDA content using commercially available assay kits (Nanjing Jiancheng, China) following the provided instructions. Furthermore, to assess the activity of antioxidant enzymes in the lung tissues, we quantified SOD and GSH levels as per the manufacturer's instructions (Nanjing Jiancheng, China).

### ROS staining of lung tissue

Frozen lung tissue sections were allowed to equilibrate to room temperature and were dried to remove any residual moisture. A histochemical pen was employed to create a boundary around the tissue to prevent the diffusion of antibodies, and an autofluorescence quencher was applied within this boundary. The sections were then rinsed under running water. Subsequently, the ROS dye solution was added meticulously within the marked boundary, and the sections were incubated in a dark incubator at 37 °C for 30 min. Following this, the slides were submerged in PBS (pH 7.4) and subjected to washing using a decolorizing shaker. After a brief air-drying step, the cell nuclei were counterstained with DAPI dye solution applied within the boundary and incubated at room temperature in the absence of light. The slides were again submerged in PBS (pH 7.4) and washed using a decolorizing shaker. Once the sections had air-dried slightly, they were mounted onto slides with anti-fluorescence quenching properties, and these slides were positioned beneath a fluorescence microscope for observation and image capture.

### Statistical analysis

The data were presented as mean values with corresponding standard deviations, and statistical analyses were carried out using GraphPad Prism 9.0 software. Group-wise comparisons were made using one-way analysis of variance (ANOVA) followed by the Student–Newman–Keuls (SNK) post hoc test. Survival analysis was performed using the log-rank test. A p-value below 0.05 was considered to indicate statistical significance.

## Results

### Liquiritigenin attenuates bleomycin-induced pulmonary fibrosis in mice

**Fig. 1:**
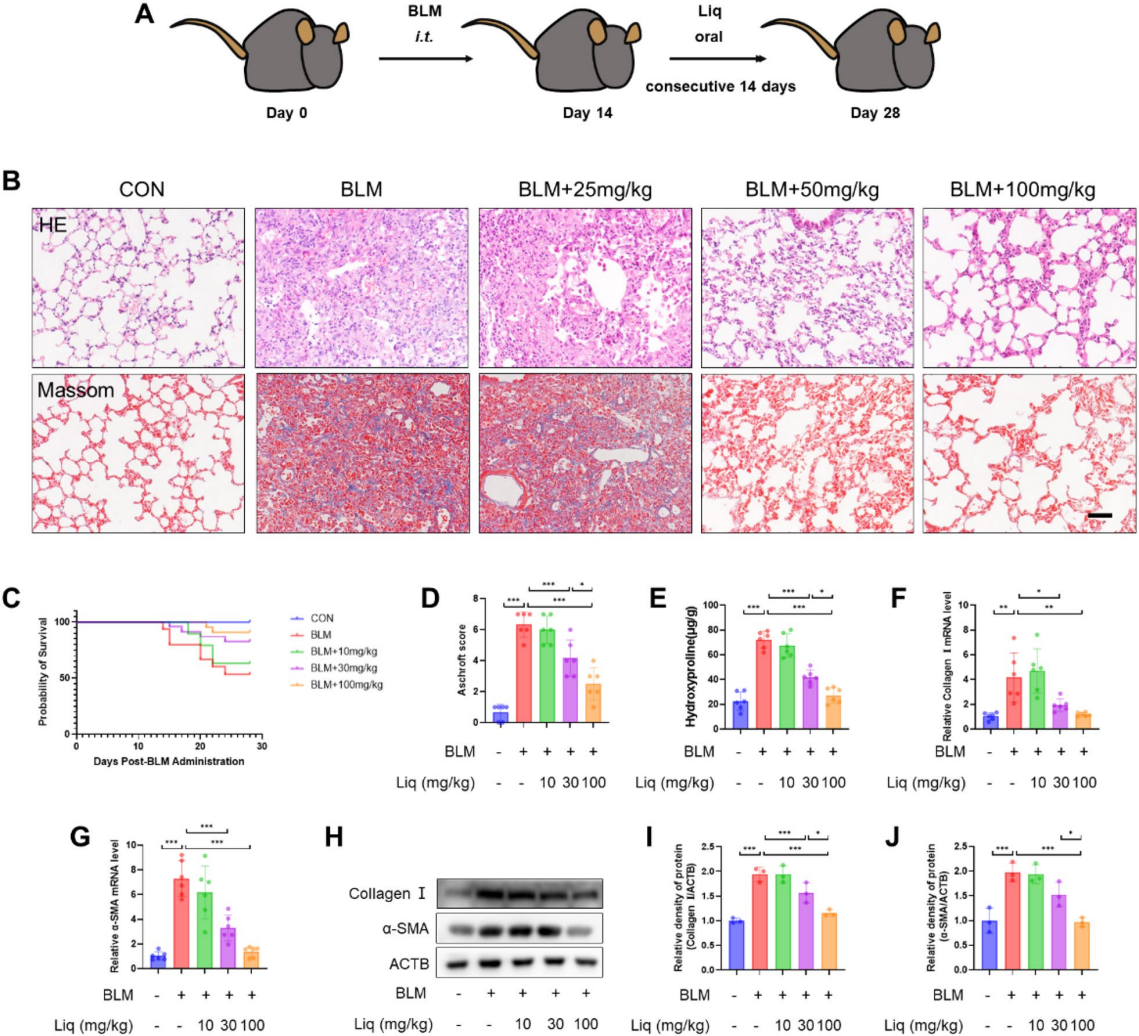
Liquiritigenin attenuates bleomycin-induced pulmonary fibrosis in mice. **A** To assess the impact of liquiritigenin on pulmonary fibrosis, mice were orally administered with 25 mg/kg, 50 mg/kg, and 100 mg/kg of liquiritigenin from day 15 to day 28 following intratracheal injection of bleomycin. **B** Lung morphology and ECM deposition were evaluated through HE and Masson staining. Scale bars represent 100 μm. **C** The survival curve of mice was recorded. **D** Scoring of pulmonary fibrosis severity in mice. **E** Hydroxyproline content, an indicator of fibrosis, was quantified using biochemical methods. **F**–**G** mRNA levels of myofibroblast markers, collagen I, and α-SMA, were analyzed through qPCR. (H-J) Protein expression levels of collagen I and α-SMA were determined by Western blotting. Data are presented as means ± standard deviation, and all experiments were independently repeated at least three times. (*P < 0.05, **P < 0.01, ***P < 0.001)

We initiated oral administration of liquiritigenin to mice from the 14th day onwards, using doses of 25, 50, and 100 mg/kg, continuously for 14 days (Fig. 1A). Notably, liquiritigenin significantly reduced the thickening of alveolar walls and deposition of extracellular matrix, with the most pronounced effect observed at the dose of 100 mg/kg (Fig. 1B, D). Furthermore, liquiritigenin dose-dependently reduced the mortality rate in bleomycin-induced pulmonary fibrosis mice (Fig. 1C). The assessment of hydroxyproline levels further confirmed the anti-fibrotic ability of liquiritigenin (Fig. 1E). Additionally, liquiritigenin inhibited the upregulation of collagen I and α-SMA, key markers of myofibroblast differentiation and critical components of fibrotic foci, which were induced by bleomycin at both mRNA and protein levels (Fig. 1F–J). In summary, liquiritigenin exhibits a concentration-dependent anti-fibrotic effect in mice with bleomycin-induced pulmonary fibrosis.

### Liquiritigenin alleviates bleomycin-induced pulmonary oxidative stress in mice

**Fig. 2:**
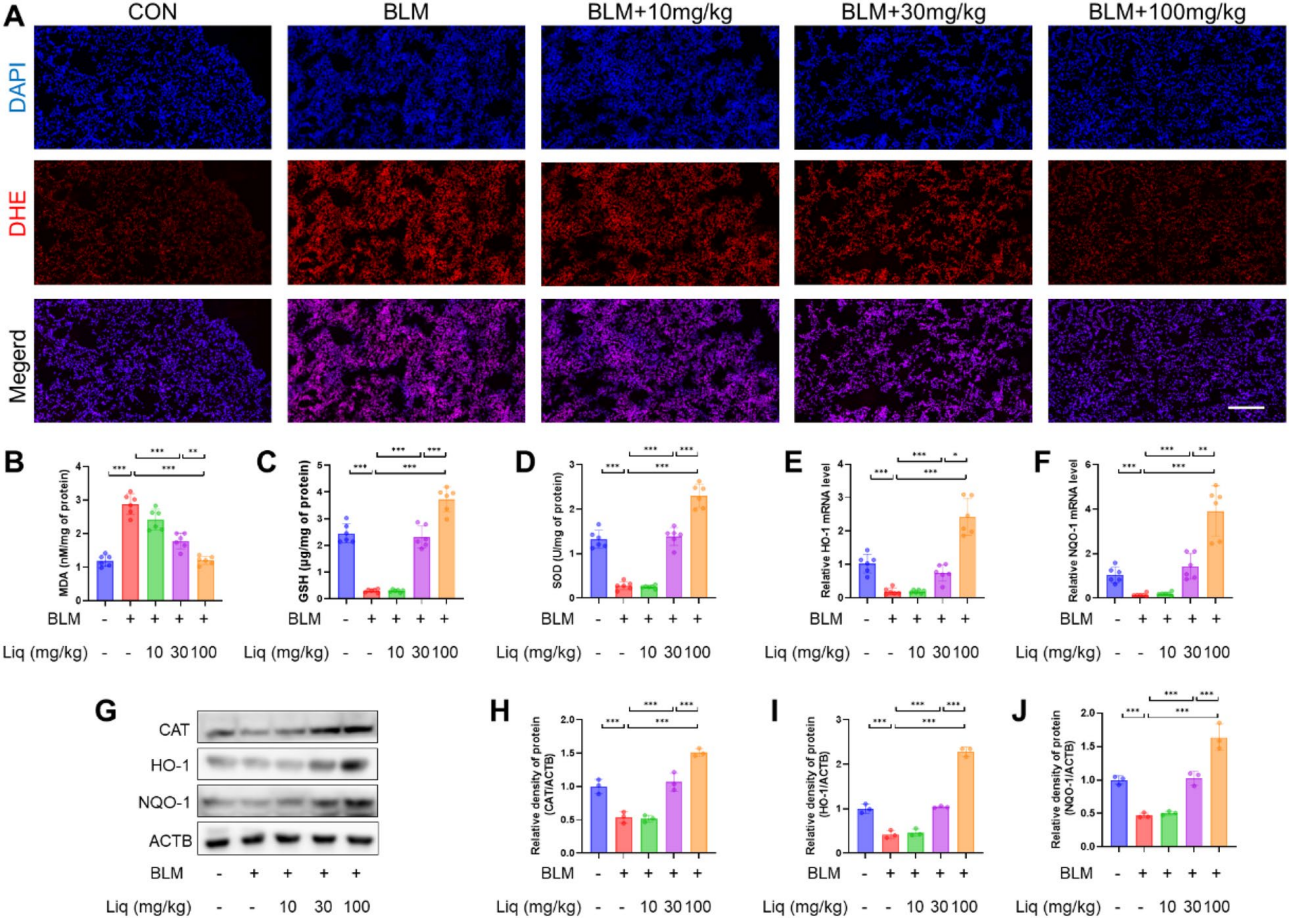
Liquiritigenin mitigates bleomycin-induced pulmonary oxidative stress in Mice. **A** DHE staining reflects lung ROS levels. Scale bars represent 100 μm. **B**–**D** Biochemical quantification of MDA and GSH levels, as well as SOD activity. **E**, **F** mRNA levels of HO-1 and NQO-1 analyzed by qPCR. **G**–**J** Protein expression levels of CAT, HO-1, and NQO-1 determined by Western blotting. Data are presented as means ± standard deviation, and all experiments were independently repeated at least three times. (*P < 0.05, **P < 0.01, ***P < 0.001)

Oxidative stress plays a crucial role in pulmonary fibrosis [7]. Therefore, we first observed that liquiritigenin reversed bleomycin-induced oxidative stress in the lungs of mice, as indicated by DHE staining (Fig. 2A). Subsequently, we assessed MDA and GSH levels, as well as SOD activity, with increasing concentrations of liquiritigenin, there was a dose-dependent reversal of the elevated MDA content, reduced GSH content, and attenuated SOD activity induced by bleomycin (Fig. 2B–D). Furthermore, liquiritigenin treatment led to a significant increase in the levels of phase II antioxidant enzymes, including CAT, HO-1, and NQO-1. (Fig. 2E–J). In summary, liquiritigenin alleviated the levels of oxidative stress induced by bleomycin in the lungs of mice.

### Liquiritigenin reverses TGF-β1-induced myofibroblast differentiation in vitro

**Fig. 3:**
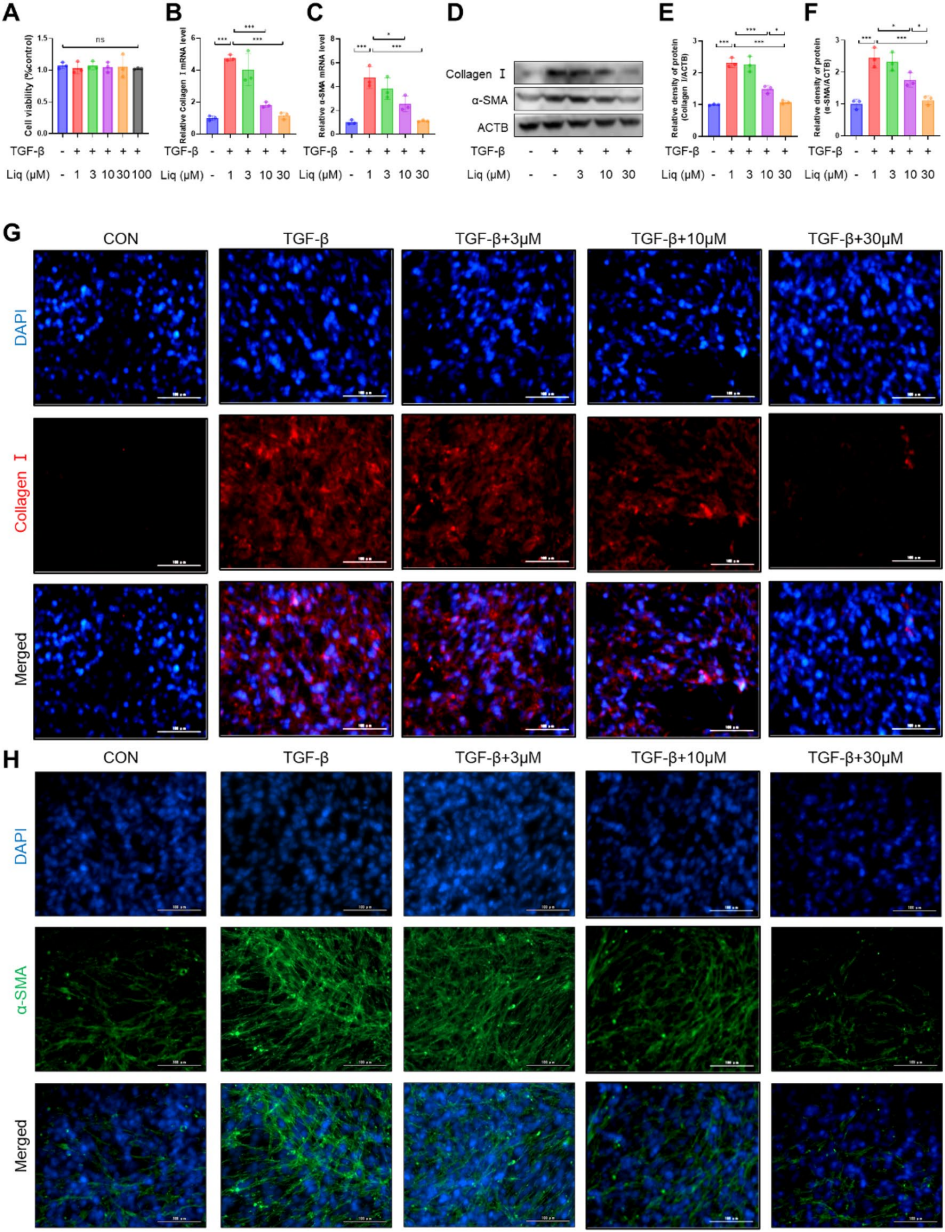
Liquiritigenin blocks TGF-β1-induced myofibroblast differentiation. **A** A CCK-8 assay was employed to determine non-toxic concentrations of liquiritigenin for myofibroblasts. **B**, **C** qPCR analysis depicted the impact of pretreatment with liquiritigenin at 3 μM, 10 μM, 30 μM, and 100 μM for 2 h on the mRNA levels of TGF-β-induced collagen I and α-SMA. **D**–**F** Protein expression levels of the myofibroblast markers collagen I and α-SMA were assessed using Western blotting. **G**–**H** Immunofluorescence microscopy visualized the effect of liquiritigenin on the TGF-β1-induced increase in collagen I and α-SMA expression levels, with scale bars representing 100 μm. Data are presented as means ± standard deviation, and each experiment was independently repeated at least three times. (*P < 0.05, **P < 0.01, ***P < 0.001)

Subsequently, we investigated the potential of liquiritigenin to inhibit TGF-β1-induced myofibroblast differentiation. First, we determined the non-toxic concentration of liquiritigenin using the CCK8 assay (Fig. 3A), and then assessed the impact of liquiritigenin pretreatment on myofibroblast markers following TGF-β1 stimulation. Both qPCR and western blotting analyses demonstrated that liquiritigenin effectively countered the TGF-β1-induced increase in collagen I and α-SMA levels (Fig. 3B–F). Immunofluorescence analysis further validated the reversal of TGF-β1-induced elevation in collagen I and α-SMA levels upon liquiritigenin treatment (Fig. 3G, H). In summary, these findings suggest that liquiritigenin effectively inhibits TGF-β1-induced myofibroblast differentiation.

### Liquiritigenin reduces TGF-β1-induced oxidative stress in vitro

**Fig. 4:**
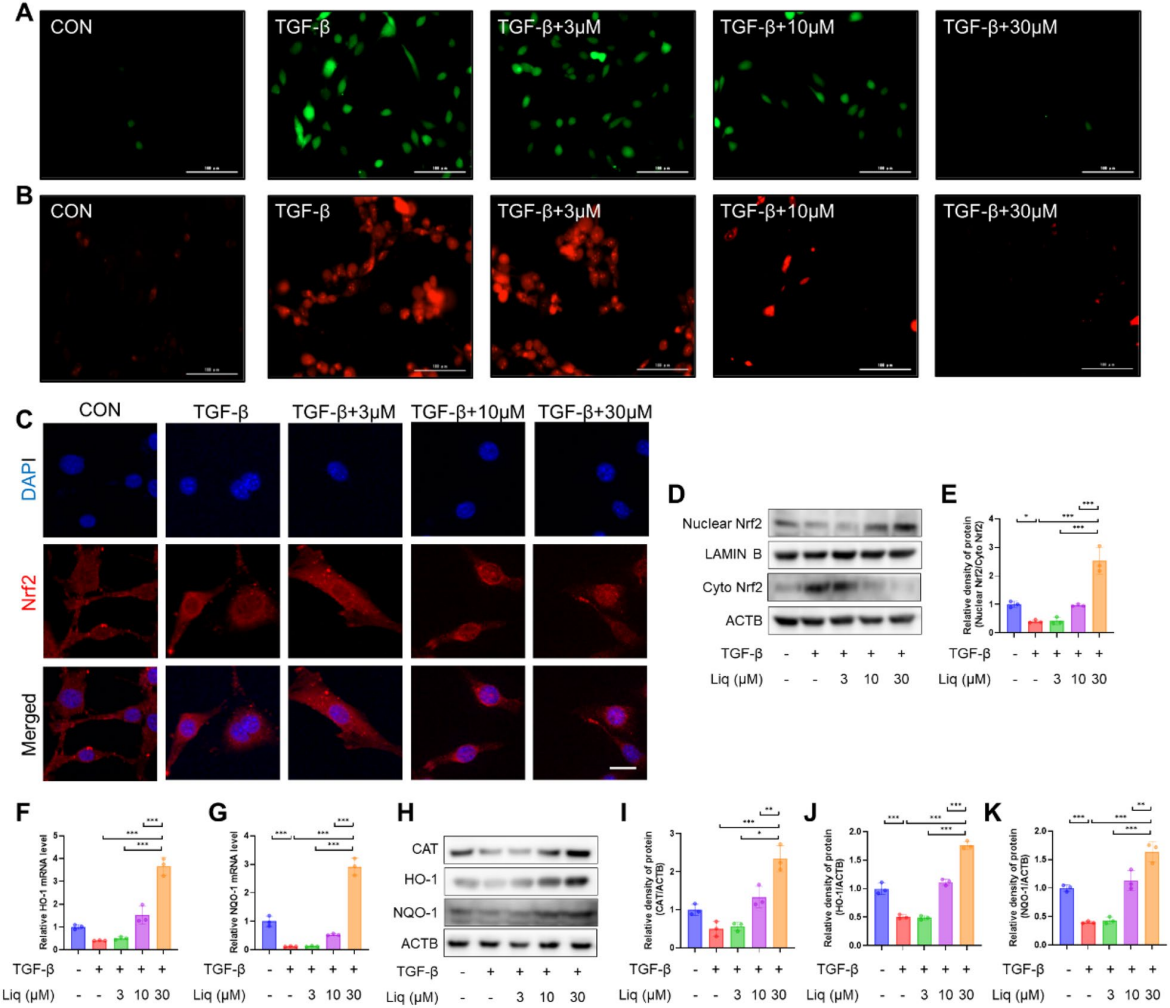
Liquiritigenin activates Nrf2 and reverses TGF-β1-induced oxidative stress. **A** H2DCFCDA probe represented ROS levels. Scale bars represent 100 μm. **B** MitoSOX probe represented mitochondrial ROS levels. Scale bars represent 100 μm. **C** Immunofluorescence microscopy observed the effect of liquiritigenin on Nrf2 nuclear translocation, with scale bars representing 50 μm. **D**–**E** The impact of liquiritigenin on Nrf2 nuclear translocation was assessed through Western blotting. **F**–**G** mRNA levels of HO-1 and NQO-1 analyzed by qPCR. **H**–**K** Protein expression levels of CAT, HO-1, and NQO-1 determined by Western blotting. Data are presented as means ± standard deviation, and each experiment was independently repeated at least three times. (*P < 0.05, **P < 0.01, ***P < 0.001)

Considering the pivotal role of ROS in fibrosis, we investigated whether liquiritigenin could counteract oxidative stress during myofibroblast differentiation. We used H2DCFDA to measure ROS levels, and the results consistently demonstrated that liquiritigenin effectively suppressed the TGF-β1-induced increase in ROS levels (Fig. 4A). Furthermore, we employed MitoSOX probes to assess levels of mitochondrial ROS, a primary source of intracellular ROS, revealing that liquiritigenin reduced mitochondrial ROS levels (Fig. 4B). Nrf2, as a regulatory target of phase II antioxidant enzymes, activates the nuclear translocation, leading to the upregulation of CAT, HO-1, and NQO-1 levels, which are crucial for ROS scavenging. The results indicated that liquiritigenin could promote Nrf2 nuclear translocation (Fig. 4C–E). Moreover, we found that liquiritigenin upregulated the mRNA and protein levels of CAT, HO-1, and NQO-1 regulated by Nrf2 (Fig. 4F–K). These findings confirm that liquiritigenin activates Nrf2 to enhance cellular antioxidant capacity, thereby alleviating TGF-β1-induced oxidative stress.

### Nrf2 mediates the blocking effect of liquiritigenin on myofibroblast differentiation

**Fig. 5:**
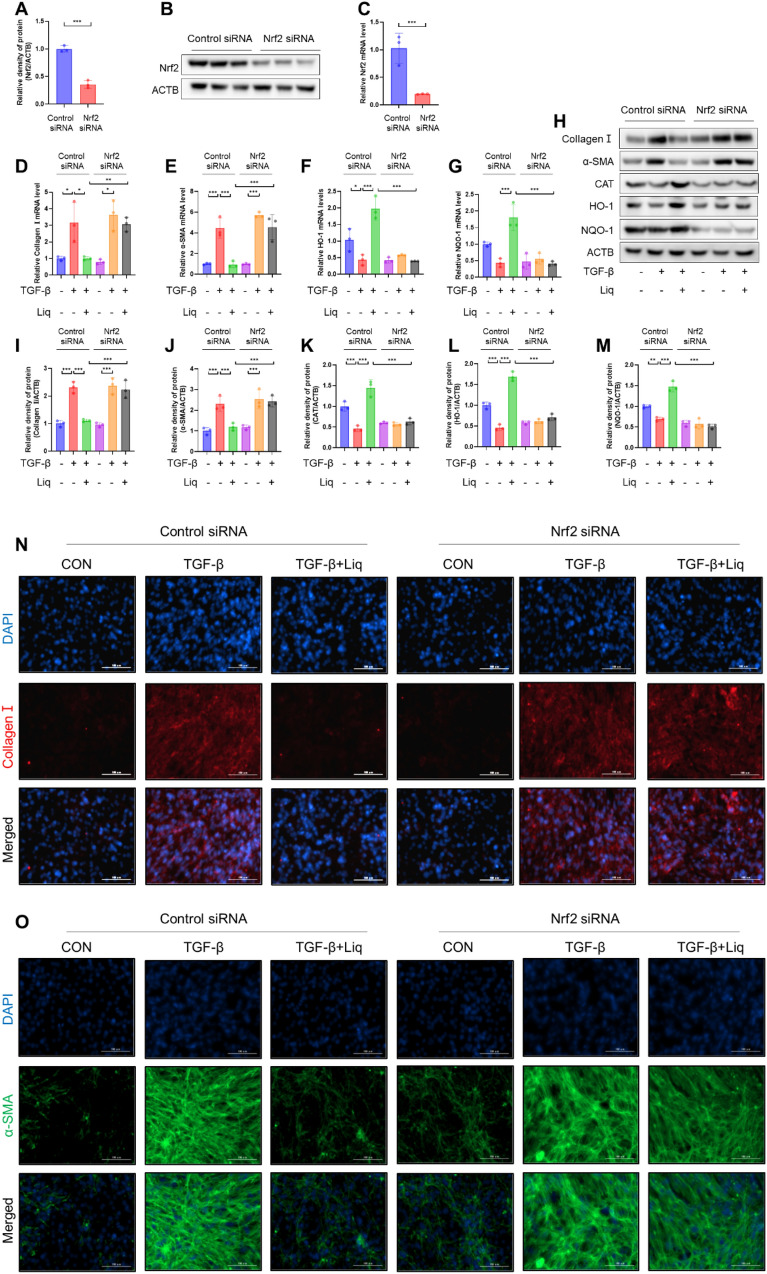
Nrf2 mediates the effects of liquiritigenin on myofibroblast differentiation. **A**–**C** Efficiency of Nrf2 siRNA was assessed by qPCR analysis and Western blotting. **D**–**G** qPCR analysis demonstrated the impact of liquiritigenin treatment on TGF-β1-induced myofibroblast collagen I, α-SMA, HO-1, and NQO-1 mRNA levels under control siRNA and Nrf2 siRNA conditions. (H-M) Western blotting assessed the protein expression levels of collagen I, α-SMA, CAT, HO-1, and NQO-1 in myofibroblasts treated with liquiritigenin under control siRNA and Nrf2 siRNA conditions following TGF-β1 intervention. **N**–**O** Immunofluorescence microscopy displayed the effect of liquiritigenin treatment on collagen I and α-SMA expression levels in myofibroblasts under control siRNA and Nrf2 siRNA conditions following TGF-β1 intervention, with scale bars representing 100 μm. Data are presented as means ± standard deviation, and each experiment was independently repeated at least three times. (*P < 0.05, **P < 0.01, ***P < 0.001)

Given the regulatory role of liquiritigenin on Nrf2 and the significant role of Nrf2 in myofibroblast differentiation, we knocked down Nrf2 expression in fibroblasts using Nrf2 siRNA (Fig. 5A–C). The results demonstrated that the knockdown of Nrf2 reversed the regulatory effects of liquiritigenin on phase II antioxidant enzymes (Fig. 5F–H, K–M). Similarly, we observed that Nrf2 silencing reversed the inhibitory effect of liquiritigenin on myofibroblast differentiation (Fig. 5D–E, H–J, N, O). In summary, our findings suggest that liquiritigenin inhibits TGF-β1-induced myofibroblast differentiation by modulating the involvement of Nrf2.

### SIRT1 regulates the liquiritigenin-mediated inhibition of myofibroblast differentiation through Nrf2

**Fig. 6:**
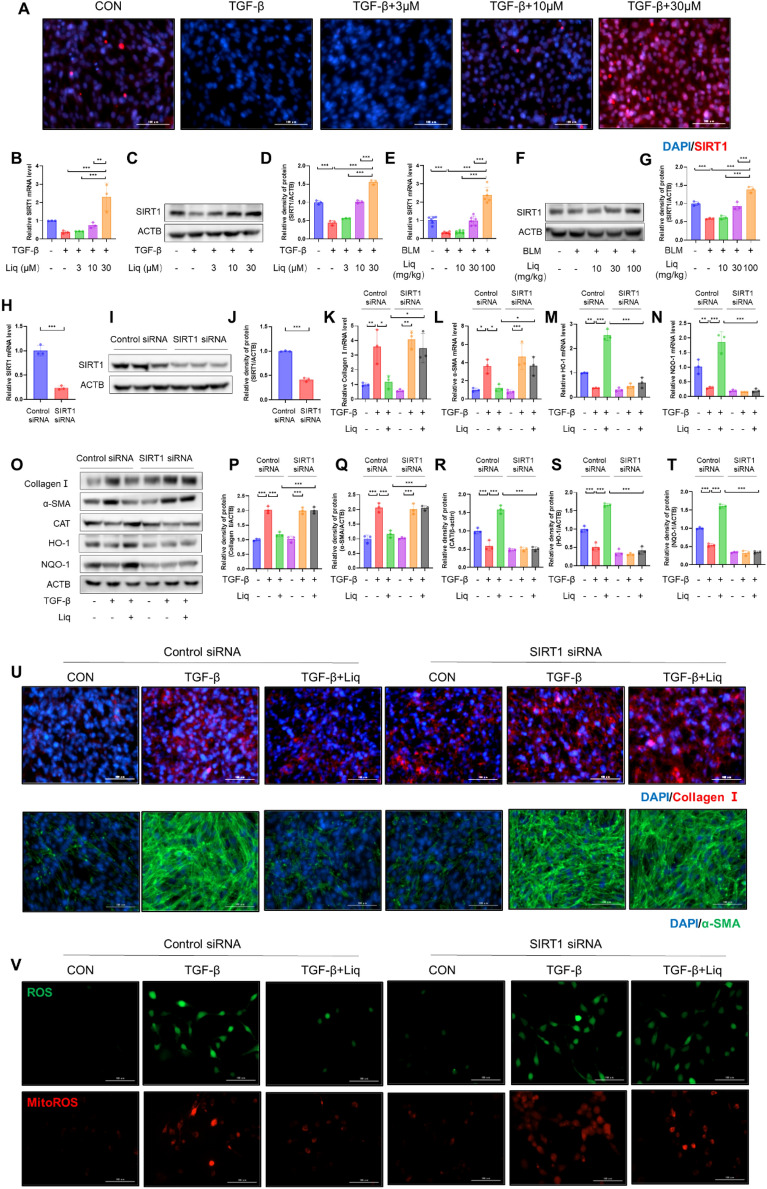
SIRT1 mediates Nrf2-induced effects of liquiritigenin on myofibroblast differentiation. **A**–**D** The influence of liquiritigenin on SIRT1 expression in vitro was evaluated through qPCR analysis and Western blotting. Scale bars represent 100 μm. **E**–**G** The impact of liquiritigenin on SIRT1 expression in vivo was assessed through qPCR analysis and Western blotting. **H**–**I** The efficiency of SIRT1 siRNA was evaluated through qPCR analysis and Western blotting. (K-N) qPCR analysis revealed the effects of liquiritigenin treatment on myofibroblast collagen I, α-SMA, HO-1, and NQO-1 mRNA levels under control siRNA and SIRT1 siRNA conditions following TGF-β1 intervention. **O**–**T** Western blotting assessed the protein expression levels of collagen I, α-SMA, CAT, HO-1, and NQO-1 in myofibroblasts treated with liquiritigenin under control siRNA and SIRT1 siRNA conditions following TGF-β1 intervention. **U** Immunofluorescence microscopy displayed the effect of liquiritigenin treatment on collagen I and α-SMA expression levels in myofibroblasts under control siRNA and SIRT1 siRNA conditions following TGF-β1 intervention, with scale bars representing 100 μm. **V** H2DCFCDA probe indicated ROS levels. MitoSOX probe indicated mitochondrial ROS levels. Scale bars represent 100 μm. Data are presented as means ± standard deviation, and each experiment was independently repeated at least three times. (*P < 0.05, **P < 0.01, ***P < 0.001)

SIRT1, as an upstream kinase of Nrf2, has been demonstrated to play a significant role in various biological activities [19]. Therefore, we investigated whether liquiritigenin could regulate the expression of SIRT1. Results from both in vitro and in vivo experiments indicated that liquiritigenin elevated the mRNA and protein levels of SIRT1 (Fig. 6A–G). Subsequently, we conducted SIRT1 knockdown in fibroblasts (Fig. 6H–J). The results demonstrated that SIRT1 knockdown reversed liquiritigenin's regulation of phase II antioxidant enzymes and ROS clearance mediated by Nrf2 (Fig. 5M–O, R–T). We also observed that SIRT1 silencing reversed the inhibitory effect of liquiritigenin on myofibroblast differentiation (Fig. 5K–L, O–Q, U). In summary, our findings suggest that liquiritigenin inhibits TGF-β1-induced myofibroblast differentiation through the SIRT1/Nrf2 signaling pathway.

### Liquiritigenin-activated SIRT1 suppresses myofibroblast differentiation regulated by the TGF-β1/Smad signaling pathway

**Fig. 7:**
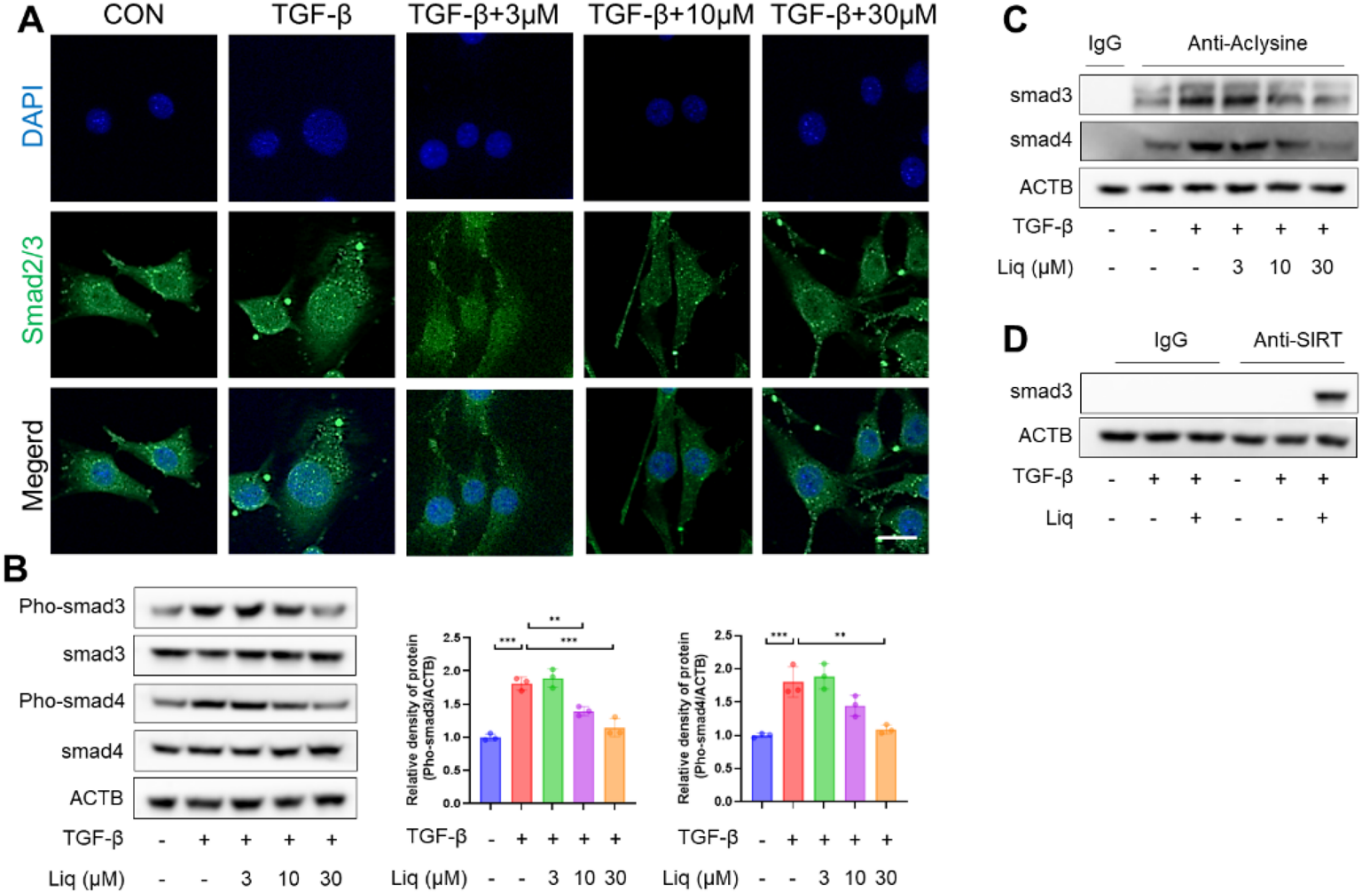
Liquiritigenin activated SIRT1 suppresses phosphorylation and acetylation of Smad. **A** Immunofluorescence microscopy displayed the effect of liquiritigenin treatment on Smad2/3 translocation in myofibroblasts with scale bars representing 100 μm. **B** Protein levels of Pho-Smad3 and Pho-Smad4 were assessed using Western blotting. **C** Deacetylation on Smad3 and Smad4 by liquiritigenin were measured by Western blotting. **D** Western blotting showed the interactions between Sirt1 and Smad3. Data are presented as means ± standard deviation, and all experiments were independently repeated at least three times. (**P < 0.01, ***P < 0.001)

Our research findings suggest that SIRT1 may directly modulate the Smad signaling pathway. In a novel discovery, we have demonstrated that during the initial phase of TGF-β1 treatment (2 h), liquiritigenin activated SIRT1. This activation, in turn, inhibits the phosphorylation and acetylation of Smad3 and Smad4, and reduced the nuclear translocation of Smad3 (Fig. 7A–C). Importantly, our study has revealed a direct interaction between SIRT1 and the Smad complex during myofibroblast differentiation (Fig. 7D). Based on our experimental evidence, we propose that the interaction between SIRT1 and the Smad complex serves as a mechanism through which activated SIRT1 effectively inhibits the Smad signaling pathway.

### SIRT1/Nrf2 pathway mediates antifibrotic effects of liquiritigenin in vivo

**Fig. 8:**
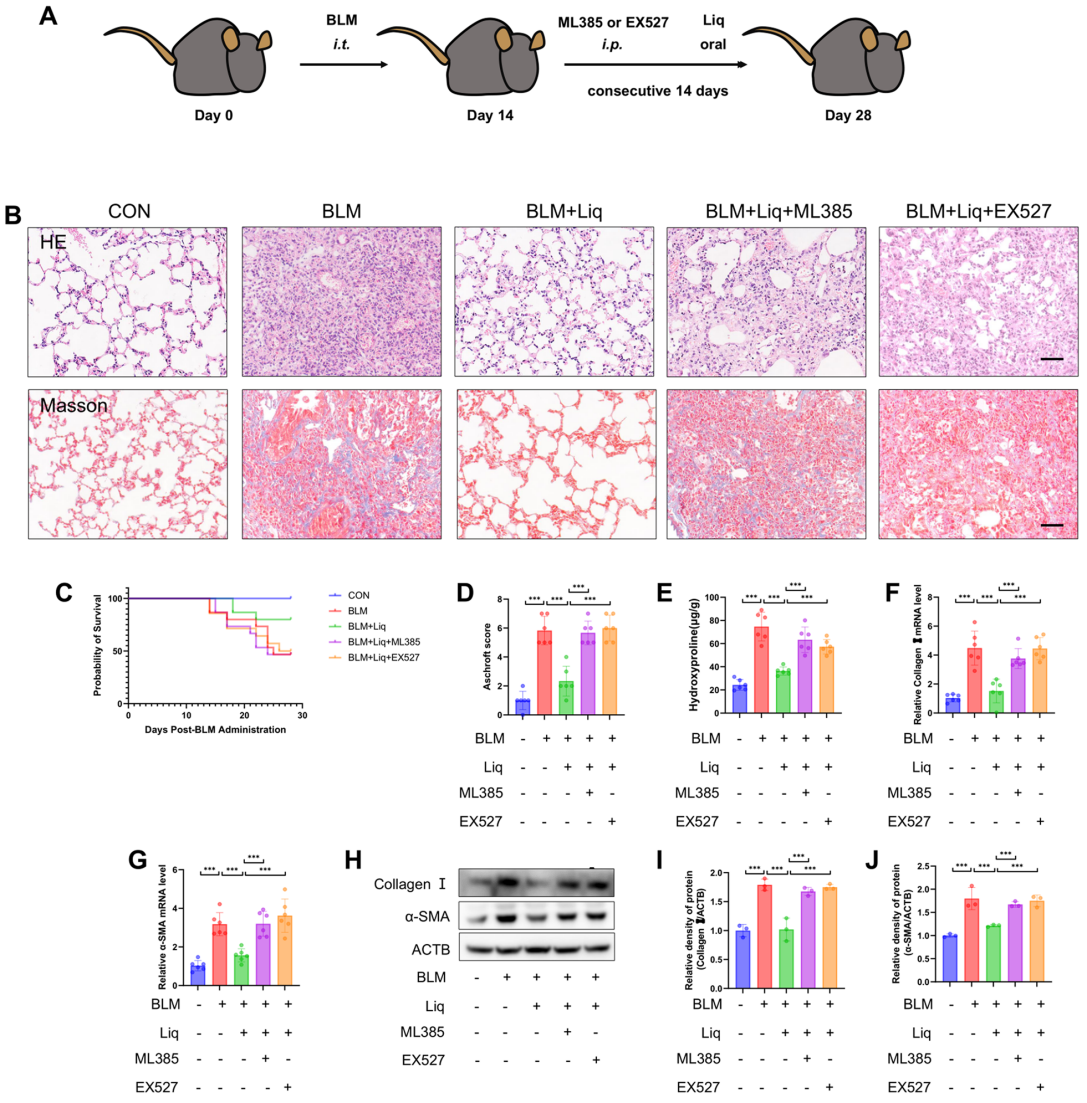
The SIRT1/Nrf2 signaling pathway mediates the effects of liquiritigenin on bleomycin-induced pulmonary fibrosis in mice. **A** To assess whether EX527 and ML385 influence the effects of liquiritigenin on pulmonary fibrosis induced by bleomycin, mice were orally administered with 100 mg/kg of liquiritigenin 3 h before receiving EX527 and ML385 from day 15 to day 28 following intratracheal injection of bleomycin. **B** Lung morphology and ECM deposition were evaluated through HE and Masson staining. Scale bars represent 100 μm. **C** The survival curve of mice was recorded. **D** Scoring of pulmonary fibrosis severity in mice. **E** Hydroxyproline content, an indicator of fibrosis, was quantified using biochemical methods. **F**–**G** mRNA levels of myofibroblast markers, collagen I and α-SMA, were analyzed through qPCR. **H**–**J** Protein expression levels of collagen I and α-SMA were determined by Western blotting. Data are presented as means ± standard deviation, and all experiments were independently repeated at least three times. (*P < 0.05, **P < 0.01, ***P < 0.001)

To validate the role of the SIRT1/Nrf2 signaling pathway in mediating the effects of liquiritigenin in bleomycin-induced pulmonary fibrosis, we administered specific inhibitors of SIRT1 (EX527) or Nrf2 (ML385) 3 h before liquiritigenin treatment (Fig. 8A). Histopathological analyses, including HE staining and Masson's trichrome staining, indicated that both EX527 and ML385 reversed the anti-fibrotic effects of liquiritigenin (Fig. 8B). This observation was further substantiated by assessing survival rates, hydroxyproline levels, collagen I, and α-SMA expression (Fig. 8C–J). In summary, our results suggest that in mice with pulmonary fibrosis, the SIRT1/Nrf2 signaling pathway mediates the anti-fibrotic effects of liquiritigenin.

### SIRT1/Nrf2 pathway mediates anti-oxidative stress effects of liquiritigenin in vivo

**Fig. 9:**
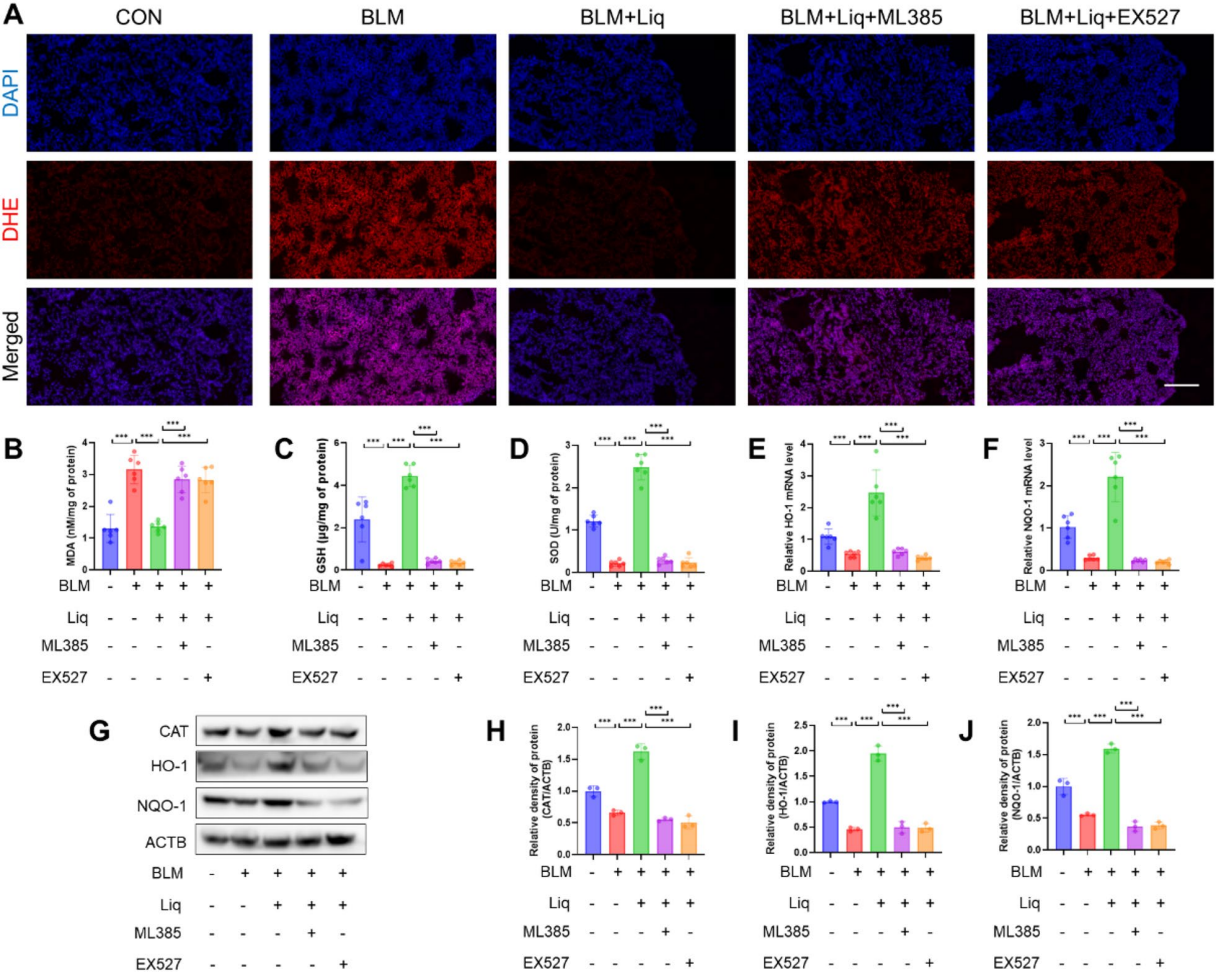
The SIRT1/Nrf2 signaling pathway mediates the effects of liquiritigenin on bleomycin-induced pulmonary oxidative stress in mice. **A** DHE staining reflects lung ROS levels. Scale bars represent 100 μm. **B**–**D** Biochemical quantification of MDA and GSH levels, as well as SOD activity. **E**, **F** qPCR analysis of HO-1 and NQO-1 mRNA levels. **G**–**J** Western blotting determined the protein expression levels of CAT, HO-1, and NQO-1. Data are presented as means ± standard deviation, and all experiments were independently repeated at least three times. (*P < 0.05, **P < 0.01, ***P < 0.001)

DHE staining revealed that EX527 or ML385 reversed the antioxidant stress effect of liquiritigenin (Fig. 9A). This observation was further supported by assessing MDA levels, GSH levels, SOD activity, as well as the expression of CAT, HO-1, and NQO-1 (Fig. 9B–J). In summary, our study results indicate that in mice with pulmonary fibrosis, the SIRT1/Nrf2 signaling pathway mediates the antioxidant stress effect of liquiritigenin.

## Discussion

Our study, for the first time, confirms that liquiritigenin can alleviate bleomycin-induced pulmonary fibrosis in mice and provides in vitro evidence that liquiritigenin could inhibit TGF-β-induced fibroblasts to myofibroblasts differentiation, the effect may be mediated by the SIRT1/Nrf2 signaling pathway.

Tracheal bleomycin injection is a critical method for establishing an IPF model. In the first 7 days following injection, the lungs undergo acute injury and epithelial cell damage, accompanied by a surge in inflammation. Subsequently, the inflammation gradually subsides and persists until day 14. From day 14 to day 28, the lungs experience a transformation of fibroblasts into myofibroblasts, marked by substantial secretion of extracellular matrix, with fibrous foci peaking on day 28 [28]. Some studies choose to initiate drug interventions from day 1, primarily for their anti-inflammatory effects rather than their direct anti-fibrotic effects [29, 30]. Focusing on the potential effect in anti-fibrosis, we opted to administer liquiritigenin orally from day 15 to day 28 and showed that orally administration of liquiritigenin alleviated bleomycin-induced pulmonary fibrosis, indicating that it may directly counteract the development of lung fibrosis.

Reportedly, oxidative stress plays a crucial role in promoting fibrosis development, including exacerbating macrophage inflammation, inducing epithelial cell apoptosis, and facilitating myofibroblast differentiation [7, 31–33]. The importance of antioxidants in pulmonary fibrosis has been emphasized, deletion of antioxidant defenses, including CAT, GSH, SOD and Nrf2 has been revealed in animal models of pulmonary fibrosis [34]. Here, we found that oral administration of liquiritigenin led to reduced levels of MDA and increased GSH levels, along with enhanced SOD activity. Additionally, it promoted the expression of downstream Nrf2-regulated phase II antioxidant enzymes such as CAT, HO-1, and NQO-1. These results suggest that the antifibrotic properties of liquiritigenin may be closely related to its antioxidative effects. TGF-β1, a key cytokine promoting myofibroblast differentiation, is regulated by oxidative stress in its secretion and activation [35]. In our in vitro experiments, liquiritigenin also activated Nrf2 in TGF-β1 stimulated fibroblasts, exhibiting its antioxidative characteristics by reducing both total ROS and mitochondrial ROS levels. Notably, myofibroblasts are recognized as the primary generators of ROS, and the release of ROS can, in turn, modulate macrophage inflammation storms and epithelial cell apoptosis [36]. Therefore, liquiritigenin's potential to mitigate ROS-mediated macrophage inflammation and epithelial cell apoptosis could be a prospective antifibrotic mechanism worthy of further investigation. Moreover, as an activator of Nrf2, liquiritigenin merits further research in other oxidative stress-mediated diseases.

SIRT1, a crucial NAD + -dependent deacetylase, plays a pivotal role in combating or delaying pulmonary fibrosis while also serving as a key antioxidant [14]. Previous research indicates that the absence of SIRT1 in organs results in elevated levels of ROS [37, 38]. Furthermore, SIRT1 has the ability to upregulate the expression of antioxidant enzymes through various pathways, thereby protecting the body from the harmful effects of oxidative stress [39]. These pathways may involve Nrf2 signaling, FOXO3A signaling, and several others [40, 41]. Consistent with these research findings, our study demonstrates that liquiritigenin significantly increases SIRT1 expression and exerts anti-fibrotic effects, the regulation of oxidative stress by liquiritigenin may involve SIRT1-mediated activation of the Nrf2 pathway. Additionally, our in vivo experiments using EX527 and ML385 further support the notion that liquiritigenin 's antioxidant effects may be mediated by the SIRT1/Nrf2 signaling pathway. Nevertheless, it is essential to note that further research is needed to elucidate the specific regulatory mechanisms between SIRT1 and Nrf2. SIRT1 plays crucial roles in the biological functions of various cell types, including endothelial cells, epithelial cells, and macrophages [13, 17]. This study primarily focuses on fibroblasts, which are pivotal in the development of lung fibrosis. Further investigation is warranted to determine whether liquiritigenin also exerts antioxidant and anti-fibrotic effects through other cell types.

TGF-β, while playing a central role in the fibrotic process, modulates ROS generation, ECM production and myofibroblast differentiation, led us to investigate the impact of liquiritigenin on the canonical TGF-β1/Smad signaling pathway [42]. Upon stimulation by TGF-β1, downstream Smad3 and Smad4 undergo phosphorylation and translocate into the cell nucleus, collectively regulating the transcription of profibrotic factors [43]. Recent studies have revealed that TGF-β1 can also induce acetylation of Smad3 and Smad4, enhancing their nuclear transcription under the catalysis of p300/CBP [44]. Activated SIRT1, a crucial target within the cellular deacetylase family, has been shown to exert deacetylation functions [45]. In line with these findings, our research demonstrates that liquiritigenin activates SIRT1, leading to the deacetylation of Smad3 and Smad4, ultimately affecting the differentiation of myofibroblasts. Importantly, our study findings indicate that activated SIRT1 directly interacts with Smad3, influencing the phosphorylation and nuclear translocation of both Smad3 and Smad4. This interaction may involve activated SIRT1 competitively binding to the phosphorylation sites of the Smad complex, thereby exerting an anti-fibrotic effect. These discoveries suggest that liquiritigenin's activation of SIRT1 directly intervenes in the TGF-β1/Smad signaling pathway, contributing to its anti-fibrotic effects. This mechanism provides a new perspective for understanding other diseases based on the Smad signaling pathway.

Our research has provided significant insights into the potential of liquiritigenin as a therapeutic agent for pulmonary fibrosis. It's worth noting that liquiritigenin serves not only as an antifibrotic but also as an antioxidative agent. Liquiritigenin can activate and enhance the expression of SIRT1, on one hand, elevating the SIRT1/Nrf2 signaling pathway to exert its antioxidative capacity and inhibit oxidative stress-mediated myofibroblast differentiation. The remarkable effects of liquiritigenin have prompted further exploration of its potential impact on other fibrosis-related pathways and diseases associated with myofibroblast differentiation. This discovery marks a significant step forward in the development of novel interventions against pulmonary fibrosis.

## Conclusions

Our findings indicated liquiritigenin’s anti-pulmonary fibrosis effect, further, its effect was mediated by the SIRT1/Nrf2 signaling pathway. Implying that targeting myofibroblast differentiation via the SIRT1/Nrf2 signaling pathway may constitute a pivotal strategy for liquiritigenin-based therapy against pulmonary fibrosis.

## Data Availability

The data used to support the findings of this study are available from the corresponding author upon request.

## Abbreviations

IPF: Idiopathic pulmonary fibrosis
TGF-β: Transforming growth factor-β
ECM: Extracellular matrix
SIRT1: Sirtuin1
*i.t.*: Intratracheal injection
*i.p.*: Intraperitoneal injection
α-SMA: α-Smooth muscle actin
Nrf2: Nuclear factor erythroid 2-related factor 2
HO-1: Heme oxygenase-1
NQO-1: Quinone oxidoreductase 1
CAT: Catalase
ROS: Reactive oxygen species

## References

[1] King TE, Pardo A, Selman M (2011). Idiopathic pulmonary fibrosis. Lancet (London, England).

[2] Chanda D, Otoupalova E, Smith SR, Volckaert T, De Langhe SP, Thannickal VJ (2019). Developmental pathways in the pathogenesis of lung fibrosis. Mol Aspects Med.

[3] Somogyi V, Chaudhuri N, Torrisi SE, Kahn N, Müller V, Kreuter M (2019). The therapy of idiopathic pulmonary fibrosis: what is next?. Eur Respir Rev..

[4] Mittler R (2017). ROS are good. Trends Plant Sci.

[5] Forrester SJ, Kikuchi DS, Hernandes MS, Xu Q, Griendling KK (2018). Reactive oxygen species in metabolic and inflammatory signaling. Circ Res.

[6] Zorov DB, Juhaszova M, Sollott SJ (2014). Mitochondrial reactive oxygen species (ROS) and ROS-induced ROS release. Physiol Rev.

[7] Otoupalova E, Smith S, Cheng G, Thannickal VJ (2020). Oxidative stress in pulmonary fibrosis. Compr Physiol.

[8] Larson-Casey JL, He C, Carter AB (2020). Mitochondrial quality control in pulmonary fibrosis. Redox Biol.

[9] Ornatowski W, Lu Q, Yegambaram M, Garcia AE, Zemskov EA, Maltepe E, Fineman JR, Wang T, Black SM (2020). Complex interplay between autophagy and oxidative stress in the development of pulmonary disease. Redox Biol.

[10] Wang Y, Wei J, Deng H, Zheng L, Yang H, Lv X (2022). The role of nrf2 in pulmonary fibrosis: molecular mechanisms and treatment approaches. Antioxidants (Basel, Switzerland)..

[11] Kikuchi N, Ishii Y, Morishima Y, Yageta Y, Haraguchi N, Itoh K, Yamamoto M, Hizawa N (2010). Nrf2 protects against pulmonary fibrosis by regulating the lung oxidant level and Th1/Th2 balance. Respir Res.

[12] Artaud-Macari E, Goven D, Brayer S, Hamimi A, Besnard V, Marchal-Somme J, Ali ZE, Crestani B, Kerdine-Römer S, Boutten A, Bonay M (2013). Nuclear factor erythroid 2-related factor 2 nuclear translocation induces myofibroblastic dedifferentiation in idiopathic pulmonary fibrosis. Antioxid Redox Signal.

[13] Imperatore F, Maurizio J, Vargas Aguilar S, Busch CJ, Favret J, Kowenz-Leutz E, Cathou W, Gentek R, Perrin P, Leutz A, Berruyer C, Sieweke MH (2017). SIRT1 regulates macrophage self-renewal. EMBO J.

[14] Chen C, Zhou M, Ge Y, Wang X (2020). SIRT1 and aging related signaling pathways. Mech Ageing Dev.

[15] Qin Z, Fang X, Sun W, Ma Z, Dai T, Wang S, Zong Z, Huang H, Ru H, Lu H, Yang B, Lin S, Zhou F, Zhang L (2022). Deactylation by SIRT1 enables liquid-liquid phase separation of IRF3/IRF7 in innate antiviral immunity. Nat Immunol.

[16] Patel S, Khan H, Majumdar A (2022). Crosstalk between Sirtuins and Nrf2: SIRT1 activators as emerging treatment for diabetic neuropathy. Metab Brain Dis.

[17] Liang J, Huang G, Liu X, Taghavifar F, Liu N, Wang Y, Deng N, Yao C, Xie T, Kulur V, Dai K, Burman A, Rowan SC, Weigt SS, Belperio J, Stripp B, Parks WC, Jiang D, Noble PW (2022). The ZIP8/SIRT1 axis regulates alveolar progenitor cell renewal in aging and idiopathic pulmonary fibrosis. J Clin Invest.

[18] Han X, Ding C, Sang X, Peng M, Yang Q, Ning Y, Lv Q, Shan Q, Hao M, Wang K, Wu X, Zhang H, Cao G (2022). Targeting Sirtuin1 to treat aging-related tissue fibrosis: from prevention to therapy. Pharmacol Ther.

[19] Mazumder S, Barman M, Bandyopadhyay U, Bindu S (2020). Sirtuins as endogenous regulators of lung fibrosis: a current perspective. Life Sci.

[20] Zhou J, Chen H, Wang Q, Chen S, Wang R, Wang Z, Yang C, Chen A, Zhao J, Zhou Z, Mao Z, Zuo G, Miao D, Jin J (2022). Sirt1 overexpression improves senescence-associated pulmonary fibrosis induced by vitamin D deficiency through downregulating IL-11 transcription. Aging Cell.

[21] Liu ZH, Zhang Y, Wang X, Fan XF, Zhang Y, Li X, Gong YS, Han LP (2019). SIRT1 activation attenuates cardiac fibrosis by endothelial-to-mesenchymal transition. Biomed Pharmacother..

[22] Ma Z, Sheng L, Li J, Qian J, Wu G, Wang Z, Zhang Y (2022). Resveratrol alleviates hepatic fibrosis in associated with decreased endoplasmic reticulum stress-mediated apoptosis and inflammation. Inflammation.

[23] Xiao Z, Chen C, Meng T, Zhang W, Zhou Q (2016). Resveratrol attenuates renal injury and fibrosis by inhibiting transforming growth factor-β pathway on matrix metalloproteinase 7. Exp Biol Med (Maywood).

[24] Lee EH, Park KI, Kim KY, Lee JH, Jang EJ, Ku SK, Kim SC, Suk HY, Park JY, Baek SY, Kim YW (2019). Liquiritigenin inhibits hepatic fibrogenesis and TGF-β1/Smad with Hippo/YAP signal. Phytomedicine.

[25] Zhang M, Xue Y, Zheng B, Li L, Chu X, Zhao Y, Wu Y, Zhang J, Han X, Wu Z, Chu L (2021). Liquiritigenin protects against arsenic trioxide-induced liver injury by inhibiting oxidative stress and enhancing mTOR-mediated autophagy. Biomed Pharmacother..

[26] Carnovali M, Banfi G, Mariotti M (2020). Liquiritigenin reduces osteoclast activity in zebrafish model of glucocorticoid-induced osteoporosis. J Pharmacol Sci.

[27] Liang F, Zhang H, Gao H, Cheng D, Zhang N, Du J, Yue J, Du P, Zhao B, Yin L (2021). Liquiritigenin decreases tumorigenesis by inhibiting DNMT activity and increasing BRCA1 transcriptional activity in triple-negative breast cancer. Exp Biol Med (Maywood).

[28] Kolb P, Upagupta C, Vierhout M, Ayaub E, Bellaye PS, Gauldie J, Shimbori C, Inman M, Ask K, Kolb MRJ (2020). The importance of interventional timing in the bleomycin model of pulmonary fibrosis. Eur Respirat J..

[29] Rui Y, Han X, Jiang A, Hu J, Li M, Liu B, Qian F, Huang L (2022). Eucalyptol prevents bleomycin-induced pulmonary fibrosis and M2 macrophage polarization. Eur J Pharmacol.

[30] Zheng F, Wu X, Zhang J, Fu Z, Zhang Y (2022). Sevoflurane reduces lipopolysaccharide-induced apoptosis and pulmonary fibrosis in the RAW264.7 cells and mice models to ameliorate acute lung injury by eliminating oxidative damages. Redox Rep.

[31] Wang Y, Zhang L, Wu GR, Zhou Q, Yue H, Rao LZ, Yuan T, Mo B, Wang FX, Chen LM, Sun F, Song J, Xiong F, Zhang S, Yu Q, Yang P, Xu Y, Zhao J, Zhang H, Xiong W, Wang CY (2021). MBD2 serves as a viable target against pulmonary fibrosis by inhibiting macrophage M2 program. Sci Adv.

[32] Lehmann M, Korfei M, Mutze K, Klee S, Skronska-Wasek W, Alsafadi HN, Ota C, Costa R, Schiller HB, Lindner M, Wagner DE, Günther A, Königshoff M (2017). Senolytic drugs target alveolar epithelial cell function and attenuate experimental lung fibrosis ex vivo. Eur Respirat J..

[33] Xie N, Tan Z, Banerjee S, Cui H, Ge J, Liu RM, Bernard K, Thannickal VJ, Liu G (2015). Glycolytic Reprogramming in Myofibroblast Differentiation and Lung Fibrosis. Am J Respir Crit Care Med.

[34] Rogliani P, Calzetta L, Cavalli F, Matera MG, Cazzola M (2016). Pirfenidone, nintedanib and N-acetylcysteine for the treatment of idiopathic pulmonary fibrosis: a systematic review and meta-analysis. Pulm Pharmacol Ther.

[35] Bellocq A, Azoulay E, Marullo S, Flahault A, Fouqueray B, Philippe C, Cadranel J, Baud L (1999). Reactive oxygen and nitrogen intermediates increase transforming growth factor-beta1 release from human epithelial alveolar cells through two different mechanisms. Am J Respir Cell Mol Biol.

[36] Barnes JL, Gorin Y (2011). Myofibroblast differentiation during fibrosis: role of NAD(P)H oxidases. Kidney Int.

[37] Long JK, Dai W, Zheng YW, Zhao SP (2019). miR-122 promotes hepatic lipogenesis via inhibiting the LKB1/AMPK pathway by targeting Sirt1 in non-alcoholic fatty liver disease. Mol Med (Cambridge, Mass).

[38] Singh V, Ubaid S (2020). Role of silent information regulator 1 (SIRT1) in regulating oxidative stress and inflammation. Inflammation.

[39] Suzuki M, Bandoski C, Bartlett JD (2015). Fluoride induces oxidative damage and SIRT1/autophagy through ROS-mediated JNK signaling. Free Radical Biol Med.

[40] Dang R, Wang M, Li X, Wang H, Liu L, Wu Q, Zhao J, Ji P, Zhong L, Licinio J, Xie P (2022). Edaravone ameliorates depressive and anxiety-like behaviors via Sirt1/Nrf2/HO-1/Gpx4 pathway. J Neuroinflammation.

[41] Chen L, Li S, Zhu J, You A, Huang X, Yi X, Xue M (2021). Mangiferin prevents myocardial infarction-induced apoptosis and heart failure in mice by activating the Sirt1/FoxO3a pathway. J Cell Mol Med.

[42] Park SA, Kim MJ, Park SY, Kim JS, Lee SJ, Woo HA, Kim DK, Nam JS, Sheen YY (2015). EW-7197 inhibits hepatic, renal, and pulmonary fibrosis by blocking TGF-β/Smad and ROS signaling. CMLS.

[43] Derynck R, Zhang YE (2003). Smad-dependent and Smad-independent pathways in TGF-beta family signalling. Nature.

[44] Inoue Y, Itoh Y, Abe K, Okamoto T, Daitoku H, Fukamizu A, Onozaki K, Hayashi H (2007). Smad3 is acetylated by p300/CBP to regulate its transactivation activity. Oncogene.

[45] Simonsson M, Kanduri M, Grönroos E, Heldin CH, Ericsson J (2006). The DNA binding activities of Smad2 and Smad3 are regulated by coactivator-mediated acetylation. J Biol Chem.

